# Guidelines for the prescription of standard hematology and biochemistry clinical laboratory tests in the intensive care unit: A scoping review protocol

**DOI:** 10.1371/journal.pone.0310059

**Published:** 2024-10-25

**Authors:** Luigi L. Devis, Emilie Catry, Michael Hardy, Alexandre Mansour, Patrick M. Honore, Giuseppe Lippi, Mélanie Closset, François Mullier

**Affiliations:** 1 Biochemistry, Department of Laboratory Medicine, CHU UCL Namur, UCLouvain, Belgium; 2 Institute for Experimental and Clinical Research (IREC), UCLouvain, Belgium; 3 Hematology, Department of Laboratory Medicine, CHU UCL Namur, UCLouvain, Belgium; 4 Department of Anesthesiology, CHU UCL Namur, UCLouvain, Belgium; 5 Namur Thrombosis and Hemostasis Center (NTHC), Namur Research Institute for Life Sciences (NARILIS), CHU UCL Namur, UCLouvain, Belgium; 6 Department of Anesthesia and Critical Care, Pontchaillou University Hospital of Rennes, Rennes, France; 7 IRSET INSERM 1085, Univ Rennes, Rennes, France; 8 Department of Intensive Care, CHU UCL Namur, UCLouvain, Belgium; 9 Section of Clinical Biochemistry and School of Medicine, University Hospital of Verona, Verona, Italy; University of Nigeria Faculty of Medical Sciences and Dentistry: University of Nigeria Faculty of Medical Sciences, NIGERIA

## Abstract

**Objective:**

This scoping review protocol describes the strategy for a scoping review that aims to provide a comprehensive overview of published guidelines for the prescription of standard laboratory tests performed in intensive care unit (ICU) patients.

**Background:**

The use of clinical laboratories is constantly increasing. However, there is evidence of inappropriate use. Inappropriate laboratory testing has the potential to harm patients, increase costs, burden staff, and has an environmental impact. Effective management can be achieved through demand managing strategies, such as providing guidelines on performing the appropriate test, for the right patient, at the right time. Although national and international guidelines exist for individual tests, a comprehensive summary of available recommendations for laboratory testing in the ICU is currently unavailable.

**Inclusion criteria:**

This scoping review will incorporate documents that provide explicit advice on which test to perform in ICU patients. We selected 34 tests routinely ordered in the ICU. This review will consider any document type that matches our concept and context. We will consider gray literature with appropriate adherence to guidelines methodology. We will not limit the review by geographical location, but will only include articles published in English.

**Search strategy:**

Our scoping review will follow the Joanna Brigg Institute (JBI) methodology. We will search Medline (PubMed), Embase, Scopus, Google Scholar, and Google. Our search strategy adheres to the JBI 3-step construction approach for systematic reviews. We will search for keywords related to guidelines, laboratory testing, and the 34 selected tests. We will report our study using the S1 Checklist.

**Review registration number:** osf.io/yfs9z.

## Introduction

Laboratory medicine is an essential component of modern healthcare and has become increasingly indispensable in clinical practice [1]. Laboratory testing enables rapid assessment of a patient physiological state, influencing most medical decisions [2]. However, inappropriate use of laboratory resources is expanding due to constant increase in laboratory utilization. It is estimated that overuse and underuse of laboratory resources occur in 20% and 40% of cases, respectively [3]. For standard hemostasis or biochemistry tests (i.e., potassium, lactate dehydrogenase, aspartate transaminase, activated partial thromboplastin time, prothrombin time/international normalized ratio) performed in high-throughput hospital laboratories, inappropriateness may span between 60% and 70% [4]. Such misuse can harm patients by causing iatrogenic anemia from frequent blood draws [5], which increases the need for transfusion and associated risks and costs [6]. It also strains staff thereby increasing error risks, causes patient discomfort and stress, raises infection risks, and may produce incidental findings leading to unnecessary further testing, diagnostic errors, and poor therapeutic decisions [3, 4, 7, 8]. Conversely, underutilization of laboratory resources can result in missed or delayed diagnoses, impacting patient safety [3]. Beyond patient care, inappropriate use of laboratory resources has also economic and environmental impacts [9, 10]. Even minor costs or small environmental impacts from individual tests or test panels can accumulate on a national scale. For example, an Australian study [11] found that an intervention aimed at reducing 10% of inappropriate routine tests ordered by internal medicine wards resulted in a reduction of 132 kg in carbon dioxide equivalent (CO_2_e) emissions and savings of AUS$ 53,000 per year. When extrapolated to all hospital wards, nationwide, the potential annual savings were estimated to be up to 135,000 kg CO_2_e and AUS$ 56 million.

Intensive care units (ICU) represent particular wards where patients require frequent blood tests. In this context, inappropriate laboratory testing is common [12, 13], leading to unnecessary additional costs [14] and harm to patients [15].

Several interventions have been published to improve the appropriateness of laboratory prescriptions. Interventions usually fall into categories such as education (raising awareness of inappropriateness and reminding clinicians about appropriate tests to request in a given clinical context), guidelines (providing recommendations on which test to perform, on which patient, at what time), audit and feedback (assessing prescription behavior and providing advice to enhance testing appropriateness), gatekeeping (restricting which test can be ordered), computerized physician order entry (interventions involving the software used to prescribe laboratory tests), and multifaceted interventions (using more than one category) [16]. In the ICU, these interventions have proven to reduce inappropriate laboratory tests and costs, while no study reported any increase in mortality, length of stay, or adverse outcome in patients [16].

Among these categories, guidelines-based strategies have proven effective [17] but require a significant effort to be implemented and maintained [18, 19]. A review of interventions conducted in the ICU setting to reduce inappropriate laboratory testing identified 44 interventions, with guidance-based methods being one of the most commonly used strategy (22/44) [16]. Surprisingly, none of the 22 studies used a national or international guideline as a model. Instead, local guidance was established through literature review and consensus among local experts. Furthermore, upon closer examination of the various guidance, discrepancies in the recommendations became apparent, underscoring the need for a comprehensive overview and systematic evaluation of the differences among the various recommendations. This is consistent with a recent systematic review that found "little consensus on best practice for reducing potentially unnecessary blood testing" [20]. Nonetheless, commendable efforts have been made to standardize appropriate laboratory testing. The Royal College of Pathologists (Britain), The Association for Clinical Biochemistry & Laboratory Medicine, and the Institute of Biomedical Science have provided guidelines for minimum retesting intervals (i.e., the minimum amount of time required between two identical tests) in 2015 [21], which were revised in 2021 [22]. In 2008, the Guidelines and Audit Implementation Network (GAIN) presented guidelines "on the Use of the Laboratory" based on data from Northern Ireland [23]. The Association for Diagnostics & Laboratory Medicine has produced a series of Laboratory Medicine Practice Guidelines (LMPGs) that cover a range of topics, including drug monitoring in pain management, tumor markers, and diabetes mellitus [24]. However, despite these efforts, a comprehensive overview of published guidance is lacking, especially in the ICU.

Some guidelines, particularly those at the national level, are not referenced in conventional medical literature databases and are considered gray literature. Furthermore, it is not the intention of our review to assess the quality of the available guidelines, but to provide a global overview of the state of the literature on tests covered by guidelines. Last, our review aims to identify potential gaps in guidelines for certain tests in the literature. Therefore, we believe that a scoping review design is better suited to capture the heterogeneity of the topic [25]. A preliminary search of PROSPERO, the Cochrane Database of Systematic Reviews, and Joanna Brigg Institute (JBI) Evidence Synthesis was conducted and no current or in-progress scoping reviews or systematic reviews on the topic were identified. The review will aim to provide a comprehensive overview of available guidelines related to laboratory test appropriateness improvement, comparing guidelines for a selected list of standard biochemistry and hematology tests, and identifying literature gaps for future reviews or guidelines.

## Methods

### Review question(s)

What are the available national or international guidelines, recommendations, or guidance are available for routinely performed hematology and clinical chemistry laboratory tests in the ICU? Are there overlapping guidelines for the same test? How consistent are different guidelines regarding the same tests? Do the guidelines offer uniform levels of recommendation? What tests lack coverage by national or international guidelines?

### Inclusion criteria

This review will consider guidelines for selected laboratory tests that are considered relevant for ICU patients (see below and Table 1).

**Table 1:** Search strategy (for PubMed) and retrieved records as of April 2024.

Search	Query	Records retrieved
#1	guidance*[ti] OR guideline*[ti] OR recommendation*[ti] OR indication*[ti] OR communication[ti]	285,614
#2	"Laboratories, Clinical"[MeSH Terms] OR "Laboratories, Hospital"[MeSH Terms] OR "Clinical Laboratory Techniques"[MeSH Terms] OR "Clinical Laboratory Services"[MeSH Terms] OR "Diagnostic Techniques and Procedures"[MeSH Terms] OR "Diagnostic Tests, Routine"[MeSH Terms] OR biomarkers[MeSH Terms] OR laborator* OR test* OR monitor* OR diagnos* OR screen*	17,030,241
#3	"Critical Care"[MeSH Terms] OR "Intensive Care Units"[MeSH Terms] OR "Intensive Care, Neonatal"[MeSH Terms] OR "Intensive Care Units, Neonatal"[MeSH Terms] OR "Intensive Care Units, Pediatric"[MeSH Terms] OR "Critical Illness"[MeSH Terms] OR ICU OR "intensive care" OR “intensive care unit*” OR "critical care" OR "critically ill" OR "critical patient"	618,616
#4	#1 AND #2 AND #3	4,812
#5	"Hematologic Tests"[MeSH Terms] OR "Blood Coagulation Tests"[MeSH Terms] OR hematology OR hemostasis OR "full blood count" OR "complete blood count" OR "blood count" OR platelet OR hemoglobin OR hematocrit OR "red blood cell*" OR "white blood cell*" OR coagulation OR "clotting screen" OR "clotting time" OR thrombophil* OR aPTT OR "activated partial thromboplastin time" OR INR OR "international normalized ratio" OR PT OR "prothrombin time" OR fibrinogen OR D-dimer* OR thrombocyto* OR thromboelasto* OR "viscoelast*" OR "ROTEM" OR “TEG” OR “Quantra” OR "PF4" OR "antiheparin-PF4 antibodies" OR “heparin-induced thrombocytopenia” OR antithrombin OR "anti-Xa" OR ADAMTS13 OR “Thrombotic thrombocytopenic pupura” OR "vitamin K antagonist*" OR heparin	6,227,511
#6	#4 AND #5	1,188
Limited to English		1,059
#8	"Blood Chemical Analysis"[MeSH Terms] OR "Clinical Enzyme Tests"[MeSH Terms] OR "Kidney Function Tests"[MeSH Terms] OR "Liver Function Tests"[MeSH Terms] OR "Pancreatic Function Tests"[MeSH Terms] OR "Thyroid Function Tests"[MeSH Terms] OR "Chemistry, Clinical"[MeSH Terms] OR "Water-Electrolyte Balance"[MeSH Terms] OR "renal panel" OR "U&E" OR electrolyte* OR urea OR BUN OR "blood urea nitrogen" OR sodium OR potassium OR chloride OR magnesium OR phosphate OR creatinine OR "liver biomarker*" OR "liver function test*" OR LFT OR AST OR "aspartate transaminase" OR "aspartate aminotransferase" OR ALT OR "alanine transaminase" OR "alanine aminotransferase" OR albumin OR "total protein" OR bilirubin OR "lactate dehydrogenase" OR "l-lactate dehydrogenase" OR LDH OR "gamma-glutamyltransferase" OR gammaglutamyltransferase OR GGT OR "alkaline phosphatase" OR ALP OR "cardiac enzyme*" OR "cardiac biomarker*" OR "creatinine phosphokinase" OR "creatine phosphokinase" OR CK OR CPK OR CKMB OR "CPK-MB" OR "CK-MB" OR troponin OR "natriuretic peptide" OR BNP OR "chemistry panel" OR "biochemistry panel" OR glucose OR CRP OR "c-reactive protein" OR "CHEM-20" OR "routine chemistry" OR lactate OR ABG OR "blood gas*" OR "arterial blood gas*" OR procalcitonin	3,240,366
#9	#4 AND #8	472
Limited to English		425

The table illustrates the search strategy employed, including the keywords and Mesh terms utilized, along with the corresponding number of hits on PubMed as of December 2023.

The core concept of our scoping review is advising on choosing the right test, for the right patient, at the right time [26], often referred to as parts of the “six-R paradigm” (i.e., Right test, for the Right patient, with the Right method, at the Right time, at the Right cost, for the Right outcome) [19]. We will specifically focus on guidelines advising on appropriate test selection for specific clinical situations.

The context of our scoping review is laboratory medicine, specifically the subfields of hematology and clinical chemistry (biochemistry) within hospital settings. Microbiology tests will not be included as microbiology represents a distinct field with its own specific analyses and laboratory workflows. Furthermore, hematology and biochemistry are particularly affected by inappropriateness. Indeed, recent literature shows a high rate of inappropriate requests in these areas, with about 60% and 70% of standard hematology and biochemistry tests, respectively, considered of doubtful clinical significance [4], likely due to increased availability and affordability of these tests through automation [27]. Finally, this review will focus on tests based on blood, which are more commonly prescribed than other matrices used in microbiology, such as urine. For these reasons, microbiology tests will not be considered. We will include tests applicable to ICU patients, and focus on those most frequently requested. We selected a total of 34 tests (13 hematology tests and 21 biochemistry tests), summarized in Table 2, based on the authors’ expertise in hematology and biochemistry laboratory medicine and within the scope of laboratory assessment functions, including screening, diagnosis, testing, and monitoring [2, 28].

**Table 2:** The 34 tests selected for the scoping review.

Hematology tests
*Conventional hematology tests*
Complete blood cell count
Platelet count
Hemoglobin
Hematocrit
*Hemostasis tests*
Activated partial thromboplastin time
Prothrombin time/International Normalized Ratio
Fibrinogen
D-dimers
Anti-Xa level/activity
Antithrombin
ADAMTS13 (a Disintegrin and Metalloproteinase with a Thrombospondin Type 1 motif, member 13) activity
Antiheparin-PF4 antibody
Viscoelastic testing (ROTEM, TEG,. . .)
Biochemistry tests
*Renal and electrolytes*
Blood urea nitrogen
Creatinine
Sodium
Potassium
Chloride
Magnesium
Calcium
Phosphate
*Liver*
Aspartate aminotransferase
Alanine aminotransferase
Gamma-glutamyltransferase
Alkaline phosphatase
Bilirubin
*Cardiac*
Cardiac troponins
Brain natriuretic peptide
Creatinine phosphokinase (isoenzyme MB)
*Other*
C-reactive protein
Glucose
Lactate
Procalcitonin
Arterial blood gas analysis

### Types of sources

We will evaluate various types of articles, such as guidelines, practice guidelines, guidance, recommendations, and consensus. We will include (i) documents officially published as guidelines by national or international organizations; (ii) guidance or recommendations from organizations, societies, or expert panels; and (iii) proposed indications by organizations, societies, or expert panels. Books, reviews, and gray literature that provide guidelines for testing will be consider if it adhere appropriately to the building methodology of guidelines. Official reports and documents from relevant conference will be reviewed if they adhere to our core concept (six-R paradigm). If a type of source fits the core concept but has been omitted from the aforementioned types, it will be included. There will be no restrictions on publication date and we will consider documents from any geographic location, provided they are in English. Additional relevant documents identified during our search will be included. We will exclude guidelines on sample collection, test execution or reporting. We will not consider comparative, descriptive or interventional studies. Abstracts, posters, oral presentations, and letters to editors will not be included.

### Search strategy

The proposed scoping review will adhere to the JBI methodology for scoping reviews [29], and in line with the Preferred Reporting Items for Systematic Reviews and Meta-Analyses extension for Scoping Reviews (PRISMA-ScR) [30].

An initial limited search of MEDLINE (PubMed) was undertaken to identify articles on the topic. The text words contained in the titles and abstracts of relevant articles, and the index terms used to describe the articles, were used to develop a full search strategy for PubMed, Embase, and Scopus (see Table 1). Separate searches for hematology and biochemistry were conducted using respective keywords. We ran successive search iterations and corrected keywords accordingly to maximize the sensitivity-specificity trade-off. In order to increase sensitivity, we will include three expert authors (one laboratory medicine specialist and two critical care specialists) in the reviewer team. However, these authors will not participate in the documents screening and data extraction. The search strategy, including all identified keywords and index terms, will be tailored for each included information source. Reference of included articles will be screened for additional documents (ascendancy approach). Articles published in English from database inception to the present will be included.

The databases to be searched include PubMed, Embase, and Scopus. Sources of unpublished studies and gray literature to be searched include Google scholar and Google. We will search Google scholar for the initial 200 articles in each of the six categories using advanced search features [31]. For Google, we will conduct a separate search for each test category using the advanced search settings and review the first 10 pages of results. To some extent, we will allow our search to be iterative as we become more acquainted with the literature. Any discrepancies with the current protocol will be reported in the final manuscript.

### Source selection

**Fig 1:**
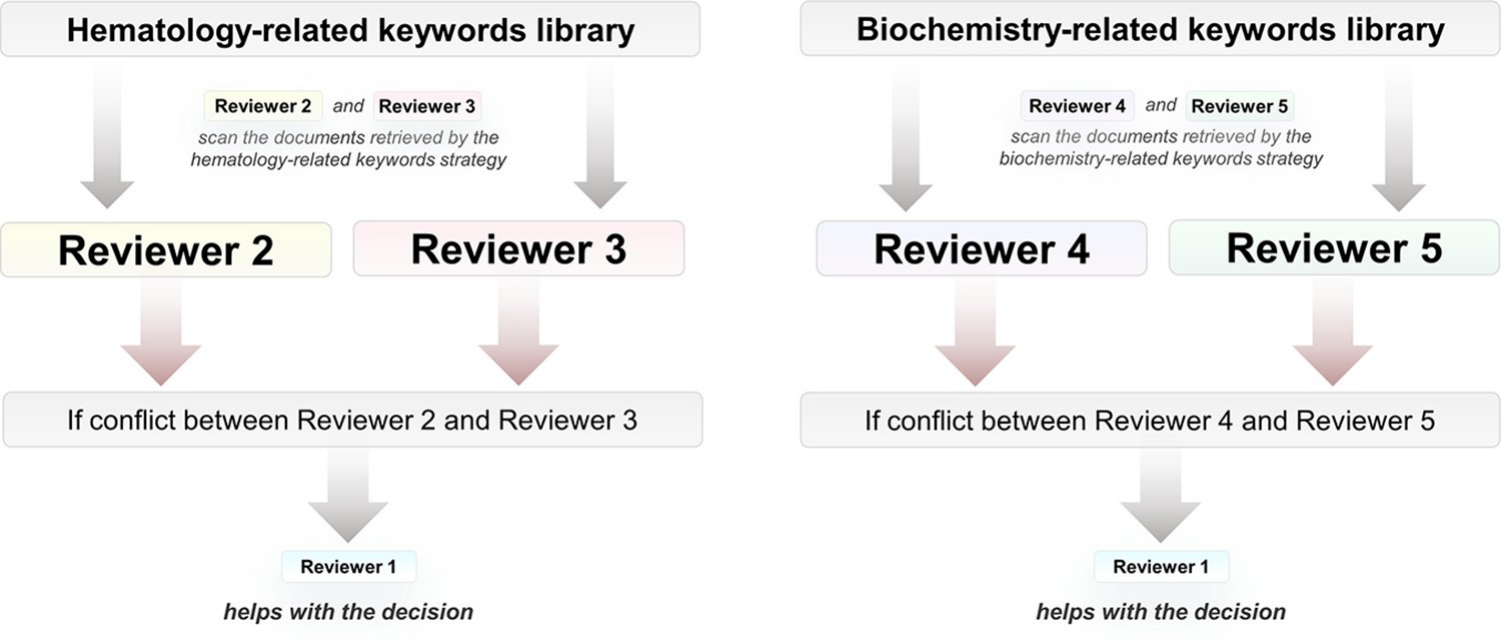
Methodology for the source selection process.

Following the search, identified records will be managed into EndNote™ 20 (Clarivate™ Analytics, PA, USA) with duplicates removed. Following a pilot test, titles and abstracts will then be screened by 4 independent reviewers for assessment against the inclusion criteria for the review, according to the following strategy (Fig 1). Two authors (Authors 2 and 3), who have an expertise in laboratory hematology, will screen the documents retrieved by the search strategy related to hematology-related keywords. Authors 4 and 5, who have expertise in laboratory biochemistry, will screen the documents retrieved by the search strategy related to biochemistry keywords. In case of any discrepancies between authors of the same subgroup, the first author (Reviewer 1) will provide management as a third party. All documents will then be screened by two independent reviewers. This approach ensures time-efficient, continuous independent screening of documents. Potentially relevant papers will be retrieved in full and their citation details imported into EndNote. Documents duplicated in both categories (hematology and biochemistry) will be reviewed by the biochemistry team. The full text of selected citations will be assessed in detail against the inclusion criteria with the same strategy. Reasons for exclusion of full-text papers that do not meet the inclusion criteria will be recorded and reported in the scoping review. The results of the search will be reported in full in the final scoping review and presented in a PRISMA flow diagram [30].

### Data extraction

Basic descriptive data will be compiled using Microsoft Excel (Microsoft Corporation, Redmond, WA, USA) including authorship type, publication year, document type, geographic location, and update status. Consistent with our review questions, we will also extract the following data (if applicable to the selected document): tests covered, qualitative description of the guidelines, number of studies informing the guidelines, and recommendation strength (if stated). In order to achieve our objectives, the document content will be extracted in a descriptive manner and the mentioned criteria will be applied to compare various guidelines for the same tests. For example, we will assess the level of agreement between different guidelines for identical tests. We will also identify the highest common factor among all the guidelines available for a particular test. In case other unforeseen data is relevant to our objectives, we will use iterations to refine our data extraction process. Any additional extracted data will be included in the final methodology description of the manuscript.

A draft extraction instrument is provided (see S1 Table). This table will undergo pilot testing on a subset of sources to assess its feasibility, and will be adapted based on feedback from the team of reviewers. The draft data extraction tool will also be modified and revised as necessary during the process of extracting data from each included article. Modifications will be detailed in the full scoping review. The data will be extracted as per the same strategy as described in the source selection process. Any disagreements that arise between the reviewers will be resolved through discussion or with a third-party reviewer.

### Data analysis and presentation

We will use basic frequency analysis and percentages as statistical descriptive tools with Microsoft Excel. We will analyze the type of authorship (e.g., expert panels, international organization), article type, publication date, and geographical location of publication in a table summarizing the data, accompanied by appropriate graphics, for all guidelines and each test category separately. The descriptive content of the guidelines will be presented in a table, and the percentage of agreement between guidelines that cover the same tests will be calculated. We will represent discrepancies and agreement in guidelines graphically. We will create maps of linked guidelines. We will visually chart the tests and categories mentioned in the guidelines to identify any possible gaps in guideline-covered tests (evidence gap maps). We will analyze and graphically present any additional data with tables or figures if deemed relevant. Any deviations from this protocol will be explicitly noted in the final manuscript of the scoping review.

## Supporting information

S1 Checklist PRIMA for systematic review protocols (PRISMA-P) checklist. (DOC)

S1 Table Data extraction instrument. Draft of the data extraction instrument that will be used for data charting. The table is susceptible to change after pilot testing.
(XLSX)
